# Whole-brain functional correlates of memory formation in mesial temporal lobe epilepsy

**DOI:** 10.1016/j.nicl.2021.102723

**Published:** 2021-06-10

**Authors:** Anna Doll, Martin Wegrzyn, Anissa Benzait, Markus Mertens, Friedrich G. Woermann, Kirsten Labudda, Christian G. Bien, Johanna Kissler

**Affiliations:** a Bielefeld University, Medical School, Department of Epileptology (Krankenhaus Mara), Maraweg 21, Bielefeld 33617, Germany; b Bielefeld University, Department of Psychology, Universitätsstraße 25, Bielefeld 33615, Germany; c Center for Cognitive Interaction Technology (CITEC), University of Bielefeld, Inspiration 1, Bielefeld 33619, Germany

**Keywords:** Episodic memory, Subsequent memory, Temporal lobe epilepsy, fMRI, AEDs, antiepileptic drugs, FWE, family-wise error rate, fMRI, functional magnetic resonance imaging, HC, healthy controls, lTLE, left temporal lobe epilepsy, lmTLE, left mesial temporal lobe epilepsy, mTL, mesial temporal lobe, mTLE, mesial temporal lobe epilepsy, perm., permutation, rTLE, right temporal lobe epilepsy, rmTLE, right mesial temporal lobe epilepsy, ROI, region of interest, SVC, small volume correction, TFCE, threshold-free cluster enhancement, TL, temporal lobe, TLE, temporal lobe epilepsy

## Abstract

The mesial temporal lobe is a key region for episodic memory. Accordingly, memory impairment is frequent in patients with mesial temporal lobe epilepsy. However, the functional relevance of potentially epilepsy-induced reorganisation for memory formation is still not entirely clear. Therefore, we investigated whole-brain functional correlates of verbal and non-verbal memory encoding and subsequent memory formation in 56 (25 right sided) mesial temporal lobe epilepsy patients and 21 controls. We applied an fMRI task of learning scenes, faces, and words followed by an out-of-scanner recognition test. During encoding of faces and scenes left and right mesial temporal lobe epilepsy patients had consistently reduced activation in the epileptogenic mesial temporal lobe compared with controls. Activation increases in patients were apparent in extra-temporal regions, partly associated with subsequent memory formation (left frontal regions and basal ganglia), and patients had less deactivation in regions often linked to the default mode and auditory networks. The more specific subsequent memory contrast indicated only marginal group differences. Correlating patients’ encoding activation with memory performance both within the paradigm and with independent clinical measures demonstrated predominantly increased contralateral mesio-temporal activation supporting intact memory performance. In left temporal lobe epilepsy patients, left frontal activation was also correlated with better verbal memory performance. Taken together, our findings hint towards minor extra-temporal plasticity in mesial temporal lobe epilepsy patients, which is in line with pre-surgical impairment and post-surgical memory decline in many patients. Further, data underscore the importance of particularly the contralateral mesial temporal lobe itself, to maintain intact memory performance.

## Highlights

• Large study of encoding and subsequent memory for words, faces, and scenes.
• Less ipsilateral mesial temporal activity in mesial temporal lobe epilepsy (mTLE).
• Extra-mTL activity in mTLE only partly relevant for memory formation.
• Across materials contralateral mTL decisive to maintain intact memory in mTLE.
• Left frontal activation correlates with better verbal memory only in left mTLE.

## 1 Introduction

In patients with temporal lobe epilepsy (TLE), memory impairments are frequent because the epileptogenic lesion and epileptic activity often disrupt brain structures involved in memory formation. (Hermann et al., 1997, Jokeit and Schacher, 2004) Some studies still support the notion that impaired verbal memory is most prominent in patients with their epileptic focus in the temporal lobe (TL) of the language-dominant hemisphere, (Hermann et al., 1987, Jeyaraj et al., 2013, Mungas et al., 1985, Saling, 2009) albeit others find that patients with their epileptic focus in the non-dominant hemisphere have equally impaired verbal memory. (Chang et al., 2019, Postma et al., 2020, Sidhu et al., 2013, Reyes et al., 2019) Impaired non-verbal memory, such as memory for line drawings and geometric figures, faces, or scenes, is even less clearly associated with an epilepsy focus in the non-language-dominant hemisphere. Rather, both right temporal lobe epilepsy (rTLE) and left temporal lobe epilepsy (lTLE) patients seem to have equally reduced non-verbal memory compared with controls. (Jeyaraj et al., 2013, Saling, 2009, Willment and Golby, 2013)

Knowledge regarding cerebral correlates of memory in TLE patients is crucial with regard to planning surgical interventions and avoiding potential adverse consequences. Moreover, data on memory formation in TLE can inform theories on cerebral correlates of memory and memory plasticity in general. Therefore, memory functions in TLE have been investigated using functional magnetic resonance imaging (fMRI) paradigms. Several studies have focused on encoding-related activity in the mesial temporal lobe (mTL). The majority of those have revealed that mTL activation is more strongly lateralised to the hemisphere contralateral to the epileptogenic focus in both rTLE and lTLE patients regardless of the encoded material (see e.g. Detre et al., 1998, Towgood et al., 2015). This has been reported to be due to reduced activation in the mTL ipsilateral to the epileptogenic focus, (Avila et al., 2006, Cheung et al., 2006, Jokeit et al., 2001, Powell et al., 2007, Sidhu et al., 2013) − but also increased activation in the mTL contralateral to the epileptogenic focus. (Figueiredo et al., 2008, Powell et al., 2007) Thus, studies indicate hypoactivation in the epileptogenic mTL, reorganisation to the contralateral side, or both. The functional relevance of increased contralateral activation is controversial: Some studies support its functionality, (Milian et al., 2015, Richardson et al., 2003, Figueiredo et al., 2008) but others suggest it is inefficient. (Powell et al., 2007, Bigras et al., 2013) In addition, several studies indicate a functional role of the ipsilateral posterior hippocampus to maintain intact, especially postoperative, memory performance. (Sidhu et al., 2013, Sidhu et al., 2015a, Bonelli et al., 2010, Bonelli et al., 2013)

Besides mTL structures, a network including lateral temporal, frontal, and parietal structures is involved in memory (for review, see Simons and Spiers, 2003). So far, few studies have examined, via whole-brain analysis, whether or to what extent TLE induces distributed changes in encoding-related activation. Extant data indicate more widespread activation in lateral temporal, frontal, and parietal regions during verbal, (Alessio et al., 2013, Dupont et al., 2000, Dupont et al., 2002, Maccotta et al., 2007, Sidhu et al., 2013) and non-verbal memory encoding, (Alessio et al., 2013, Limotai et al., 2018, Maccotta et al., 2007, Sidhu et al., 2013) in both lTLE and rTLE patients compared with controls. Overall, additional recruitment of lateral temporal regions appears most consistent. (Sidhu et al., 2013, Alessio et al., 2013) An increase in frontal activation varies both among studies and within individual studies across different encoding materials. Additional activation in frontal areas contralateral to the epileptogenic focus has been inconsistently reported during verbal and non-verbal memory encoding in either lTLE patients, rTLE patients, or both. (Sidhu et al., 2013, Alessio et al., 2013, Maccotta et al., 2007, Limotai et al., 2018) Some inconsistencies might be due to the fact that existing studies had mostly rather small sample sizes and partly lacked statistical comparison with a control group, thus potentially over-interpreting descriptive differences. Furthermore, it remains unclear if reported changes in activation are specific to memory formation or reflect other unrelated cognitive processes, because most extant whole-brain studies only focused on encoding, but did not calculate subsequent memory effects. (Maccotta et al., 2007)

The subsequent memory effect is a more specific measure of memory formation. It contrasts fMRI activation during encoding of subsequently remembered items with subsequently forgotten ones. Whereas the more general encoding activation represents a mixture of perceptual, attentional, and memory networks, subsequent memory analyses show activation specific to *successful* memory formation. Within the mTL, to our knowledge, only three studies have examined this effect in TLE patients. (Richardson et al., 2003, Powell et al., 2007, Bonelli et al., 2010) They have shown divergent effects for words and faces and consistently no difference for line drawings between a control group and lTLE or rTLE patients (for details, see Supplementary Table 1, columns 1 −3).

Two more recent studies have extended subsequent memory analyses in TLE to the whole brain (see Supplementary Table 1, columns 4 −5). Sidhu et al. (Sidhu et al., 2013) investigated the encoding of faces and words in 44 mesial temporal lobe epilepsy (mTLE) patients with hippocampal sclerosis and a control group. In controls, the verbal subsequent memory effect was restricted to left temporal and frontal regions, but lTLE patients exhibited right temporal activation. Non-verbal subsequent memory activated right temporal and frontal regions in controls, whereas rTLE patients showed right temporal and predominantly left extra-temporal activation. Regarding extra-temporal regions, subsequent memory analysis and correlations of activation and neuropsychological memory measures underline the critical involvement of the orbitofrontal cortex in both controls and mTLE patients in memory formation, whilst the insula and the anterior cingulum have been found to play a role only in mTLE patients. (Sidhu et al., 2013) Taken together, the findings by Sidhu et al. (Sidhu et al., 2013) are in line with the notion of epilepsy-induced functional reorganisation, but the authors did not report direct between-groups comparisons. Hill et al. (Hill et al., 2020) focused on verbal subsequent memory in a heterogeneous sample of five left, seven right and four bilateral TLE patients, none of whom had hippocampal sclerosis. When comparing across groups − without a statistically significant group difference − they identified the verbal subsequent memory effect in the left inferior frontal gyrus, but not in the mTL. Further, unlike what has been commonly assumed, they found no impact of the epileptogenic hemisphere (Hill et al., 2020). However, it remains unclear to what extent the lack of significant differences might be due to the specific material used or the heterogeneous and small sample.

In summary, data regarding the general encoding network predominantly indicate mTL lateralisation contralateral to the epileptogenic focus in TLE patients. Moreover, TLE patients may have increased lateral temporal activation as well as activation changes in frontal regions, but their functional relevance is unclear. Evidence is scarce for the more specific subsequent memory effect. Only a handful of studies investigated subsequent memory effects in temporal lobe epilepsy with varying results. More comprehensive knowledge about potential changes in the brain’s memory encoding system is needed. This comprehension is especially important for TLE patients who are candidates for surgical treatment. For those patients, we not only need to identify the functionality of the to-be-resected mTL area, but also understand potential compensatory mechanisms across the whole brain to better predict postsurgical memory sparing or decline.

To address these issues, we have applied a memory fMRI task of learning words, faces, and scenes. Several studies, (Avila et al., 2006, Bigras et al., 2013, Cheung et al., 2006, Detre et al., 1998, Golby et al., 2002, Limotai et al., 2018, Mechanic-Hamilton et al., 2009) have explored encoding-related activity for complex scenes in TLE, but so far none has investigated specifically the subsequent memory effect for this material. We have also optimised scanning parameters by means of coronal slices aligned with the long axis of the hippocampus to investigate mesio-temporal and extra-temporal activation in one study. This endeavour is challenging: Many studies focusing on the whole brain did not detect significant hippocampal activation. (Alessio et al., 2013, Dupont et al., 2000, Maccotta et al., 2007, Hill et al., 2020) To further engage the hippocampus via emotional modulation, taking advantage of the reciprocal connection of hippocampus and amygdala, (Strange et al., 2014) we have included a mixture of both emotional and neutral stimuli. We have performed statistical comparison of encoding- and subsequent memory-related activation between groups and further correlated the fMRI activation with memory performance in mTLE patients, including independently obtained clinical neuropsychology performance measures, in order to determine the functionality of activation also with more ecologically valid measures. Thereby, we aimed to extend knowledge on the brain basis of memory encoding and, more specifically, subsequent memory formation in mTLE patients.

## 2 Materials and methods

### 2.1 Participants

We studied 56 patients with mTLE (25 right sided), who were consecutively recruited from the pre-surgical evaluation ward at Mara Hospital, Bielefeld, Germany, and 21 healthy controls (HC). Four patients had incomplete datasets: One right mesial temporal lobe epilepsy (rmTLE) patient had a seizure during the last run (completing only words and scenes) and thus the recognition task could not be performed after scanning. For one left mesial temporal lobe epilepsy (lmTLE) patient, the fMRI data of scenes and faces could not be analysed due to technical issues during the recordings. Further, two lmTLE patients discontinued the fMRI task after the second run (one completed words and faces, the other one completed words and scenes), but both still performed the recognition task.

Patients’ epileptic onset zone was ascertained via video-EEG monitoring and structural MRI at 3 T to be unilateral and in the mTL. All patients spoke fluent German and received antiepileptic drugs (AEDs). The individual AED load was estimated as the sum of the ratios of the prescribed daily dose by the estimated average dose prescribed (“defined daily dose”) as reported by the World Health Organization (see https://www.who.int/tools/atc-ddd-toolkit/about-ddd) for each prescribed AED. (Deckers et al., 1997) Requirements for control participation were ≥18 years old and absence of known neurological and psychiatric disorders. A detailed description of all participants’ demographic and patients’ clinical characteristics is given in Table 1.

**Table 1:** Demographic and clinical characteristics of controls and lmTLE and rmTLE patients.

	Controls *n* = 21	lmTLE *n* = 31	rmTLE *n* = 25
Age in years [*M (SD)* (*Min*; *Max*)]	34.7 (12.8) (20; 60)	40.6 (14.2)# (18; 60)	37.0 (12.2) (19; 61)
Sex in % [male/female]	57.1/42.9	45.2/54.8	52.0/48.0
Years of schooling [*M (SD)* (*Min*; *Max*)]	10.6 (1.5) (9; 13)	10.7 (2.1) (4; 13)	10.6 (1.7) (9; 13)
Handedness [right/left]	19/2	28/3	25/0
Verbal learning z-score (Clinical assessment) [*Mdn* (*Min*; *Max*)]	0.8 (-1.3; 2.0)	-0.3* (-2.0; 1.6)	0.4 (-1.3; 2.0)
Design learning z-score (Clinical assessment) [*Mdn* (*Min*; *Max*)]	0.7 (-1.0; 2.0)	-0.2* (-2.5; 3.1)	-0.6* (-2.7; 2.0)
Language lateralitya) [right/left/bilateral/unknown]		0/27b)/3/1	1/21/2/1
Epilepsy onset [*M (SD)* (*Min*; *Max*)]		20.2 (12.4)^c^ (3; 55)	23.0 (12.0) (1; 57)
Years with epilepsy [*M (SD)* (*Min*; *Max*)]		20.4 (16.2)c) (0; 55)	14.0 (9.8) (0; 40)
Antiepileptic drug load [*Mdn* (*Min*; *Max*)]		2.3 (0.3; 5.3)	2.3 (0.7; 5.3)
Aetiology Hippocampal sclerosis/mesio-temporal sclerosis/tumors/cavernomas/cavernoma + hippocampal sclerosis/encephalocele + amygdala lesion/unspecified mesial lesions/no lesion		20d)/4/1/1/1/1/2/1	19e)/1/4/0/0/0/1/0

Abbreviations: lmTLE, left mesial temporal lobe epilepsy; rmTLE, right mesial temporal lobe epilepsy.

# lmTLE patients were older than controls, but the difference was not significant (*U* = 244.5, *p* = .13).

* mTLE patients had reduced memory performance compared with controls (*ps* < 0.05).

a) Language laterality was determined by the radiologist based on language fMRI for all but one patient, for whom it was determined by Wada test.

b) For two of the presumably left-lateralised patients, the assessment is uncertain.

c) For two patients epilepsy onset was estimated to have been with 3 years, as records specified it to have been “in earliest childhood”.

d) One with additional mesial cavernoma.

e) One with additional mesial heterotopia and one with additional mesial paraneoplastic encephalitis.

All participants had normal or corrected-to-normal vision. They gave written informed consent according to the Declaration of Helsinki. The University of Bielefeld’s and the Westfalen-Lippe medical association ethics committees approved the study.

### 2.2 Neuropsychological testing

All participants but one healthy control underwent neuropsychological testing including verbal and visual memory assessment. For verbal memory, the German adaption of the Rey Auditory Verbal Learning and Memory Test (Schmidt, 1996) (Verbaler Lern- und Merkfähigkeitstest (Helmstaedter et al., 2001), VLMT) was used and visual memory was assessed via the Diagnosticum for Cerebralschädigung II (Weidlich et al., 2011) (DCS), which were part of the standard diagnostic procedures during patients’ stay at the pre-surgical evaluation ward. In the VLMT participants listen to five repetitions of the same list of 15 unrelated words, which have to be freely recalled each time. In the DCS participants are similarly presented 9 geometrical figures for 10 s each and then asked to reproduce the memorised figures with wooden sticks. There are again five trials. The DCS was available for all but two lmTLE patients. For both tests the standardised, age-corrected z-scores of the sum of recalled items across five trials were calculated according to the norms of the respective test.

### 2.3 Memory-encoding paradigm

The encoding paradigm comprised three stimulus conditions, namely scenes, faces, and words. Scenes were coloured complex scenes mostly taken from the International Affective Picture System (IAPS) set, faces were front-view colour photographs of adult faces, and words were single nouns (see Supplementary Table 3 for a list of all stimuli used). Each stimulus condition included 36 negative and 36 neutral randomly selected stimuli. In three randomised consecutive runs, the stimuli of each condition were presented for 3 s each in alternating blocks of four neutral or four negative stimuli, followed by a 12-second baseline condition. Participants were instructed to encode and memorise the stimuli for the out-of-scanner recognition task. In the baseline condition, participants were asked to maintain fixation at a randomly moving dot and to avoid thinking about the preceding stimuli.

The recognition task outside of the scanner immediately followed the fMRI task, about 10 −15 min after encoding. The three stimulus conditions were presented in the same order as in the fMRI task. In each condition, all 72 previously displayed stimuli and an additional 48 new stimuli, which served as foils, were presented. Participants were asked to press the right arrow key, labelled with “yes”, for stimuli shown in the MRI and the left arrow key, labelled with “no”, for new stimuli.

### 2.4 Magnetic resonance data acquisition

MRI data were collected on a 3 T Siemens Verio MRI scanner. High-resolution T1-weighted structural data were acquired using a 32-channel head coil, with 192 sagittal slices, a slice thickness of 0.8 mm, a field of view of 15.36×24×24 cm, 0.75×0.75 mm in-plane resolution, echo time of 2.5 ms, and repetition time of 1.9 s. For fMRI, gradient-echo planar images were acquired using a 12-channel head coil, with 37 coronal slices aligned with the long axis of the hippocampus, a slice thickness of 4 mm, a field of view of 19.2×14.8×19.2 cm, 2.4×2.4 mm in-plane resolution, echo time of 33 ms, and repetition time of 3 s. Three additional dummy scans were included to ensure a steady state of magnetisation.

### 2.5 Anatomical and functional data pre-processing

MRI data were pre-processed using fMRIPrep 1.4.1rc4, (Esteban et al., 2019) which is based on Nipype 1.2.0. (Gorgolewski et al., 2011) A deformation field to correct for susceptibility distortions was estimated based on fMRIPrep’s fieldmap-less approach. The deformation field results from co-registering the fMRI reference to the same-participant T1-weighted reference with its intensity inverted. (Wang et al., 2017, Huntenburg, 2014) fMRI images were slice-time corrected using 3dTshift from Analysis of Functional NeuroImages (AFNI) 20160207 (Cox and Hyde, 1997) and resampled into MNI152NLin2009cAsym standard space. Resampling was performed using antsApplyTransforms configured with a one-step Lanczos interpolation by collapsing all the pertinent transformations (i.e. head-motion transform matrices, susceptibility distortion correction, slice-time correction, and co-registrations to MNI space) to minimise the smoothing effects of other kernels. (Lanczos, 1964)

### 2.6 Statistical analysis

#### 2.6.1 Behavioural data

We compared lmTLE and rmTLE patients and controls regarding the percentage of hits and false alarms, recognition accuracy, and response bias separately for each condition using Python 3.6.8 and the SciPy package. (Virtanen et al., 2020) We calculated recognition accuracy (hits − false alarms) and response bias (false alarms/[100 − recognition accuracy]) according to the two-high threshold model. (Snodgrass and Corwin, 1988) Because the data were partly not normally distributed (Shapiro-Wilk *p* < 0.05), we used the Mann-Whitney *U* test to compare groups. We compared behavioural data with two-tailed tests and a critical *p* value of 0.05.

#### 2.6.2 fMRI data

We conducted the first level analyses using Python 3.6.8 and the module Nistats 0.0.1b1 (https://nistats.github.io). The imaging time series were smoothed with a Gaussian kernel of 8 mm full-width at half-maximum and high-pass filtered with a cut-off at 0.01 Hz. We included the six movement parameters, frame-wise displacement, and the first six components of the anatomical-component-based noise correction (Behzadi et al., 2007) as confounding factors. For the second level analyses, we used the FMRIB Software Library (FSL) 6.0 randomise 2.9 non-parametric permutation (perm.) testing with 10,000 permutations. (Winkler et al., 2014) We added age at evaluation as a nuisance variable for the second level analysis because age slightly differed between groups. We corrected all results for multiple comparisons using threshold-free cluster enhancement (TFCE). (Smith and Nichols, 2009) The significance level was set to *p* < 0.05 family-wise error rate (FWE) corrected. We additionally display *t*-values significant at *p*_(FWE)_ < 0.1 to give a more comprehensive picture of the activation pattern. We used this threshold, instead of an uncorrected *p* value, in group comparison and correlation analyses to ensure a certain level of sensitivity while still being aware of the expected FWE rate. (Bennett et al., 2009, Roiser et al., 2016) We conducted whole-brain and small volume correction (SVC) analyses for a region of interest (ROI) encompassing the hippocampus and the amygdala as defined in the Harvard-Oxford Atlas because of the often low signal-to-noise ratio in this region. (Powell et al., 2005, Strange et al., 2002, Olman et al., 2009)

##### 2.6.2.1 Blocked analyses: Encoding scenes, faces, and words

The regressors of interest were the three stimulus categories − scenes, faces, and words, each convolved with the canonical double gamma haemodynamic response function. We created first level contrast images for these three conditions each contrasted with the respective baseline condition. Then, on the second level, we used one-sample *t*-tests to examine statistically significant group-invariant main effects of encoding elicited by each stimulus modality and two sample *t*-tests to compare mTLE patients with controls within each modality. To further investigate individual lateralisation of memory function in a combined mask with the hippocampus and amygdala during each stimulus condition, we calculated lateralisation-indices as follows: LI = [(L − R)/(L + R)], where L and R indicate the number of voxels with *z* >1.96 in the left and right mTL.

Additionally, we aimed to explore functionality of neural reorganisation in lmTLE and rmTLE patients by calculating correlations between the fMRI encoding activation of each condition with both, the respective recognition accuracy in the experiment, and either verbal or design learning scores as determined via clinical neuropsychology assessment.

##### 2.6.2.2 Event-related analyses: Subsequent memory effects of scenes, faces and words

Like previous studies, (Richardson et al., 2003, Powell et al., 2007, Bonelli et al., 2010) we also analysed the blocked fMRI task with an event-related approach. In these analyses, our regressors of interest were subsequently remembered items and subsequently forgotten ones, separately for each condition, which were contrasted for the first level analyses. We took these first level contrasts of remembered vs. forgotten items to the second level, first for a one-sample *t*-test collapsed across groups to assess regions relevant for successful memory formation in each modality and then for two-sample *t*-tests to evaluate potential reorganisation of regions relevant for successful memory formation in mTLE patients compared with controls.

Due to the wide range of recognition performances across participants, we had to limit the subsequent memory contrast to subgroups (which varied for the stimulus modalities), similarly to previous studies. (Hill et al., 2020, Clark and Wagner, 2003, Park et al., 2013) This guards against spurious estimates due to floor and ceiling effects or guessing, because some participants showed excellent or very poor performance, thus having only a very small number of either subsequently forgotten or remembered items. Again, others showed a poor recognition accuracy suggestive of guessing. All cases result in a very uncertain estimate of the subsequent memory contrast. To deal with this factor and to still achieve a valid subsequent memory contrast, we only included participants with at least 10% (eight items) of subsequently remembered and forgotten items and a recognition accuracy of at least 10%. These criteria resulted for scenes in samples of 12 controls, 25 lmTLE, and 23 rmTLE patients; for faces in samples of 18 controls, 23 lmTLE, and 17 rmTLE patients; and for words in samples of 18 controls, 27 lmTLE, and 22 rmTLE patients.

### 2.7 Data availability

All unthresholded statistical fMRI maps are available on NeuroVault (https://identifiers.org/neurovault.collection:8993). Further data from this study are available from the corresponding author upon reasonable request.

## 3 Results

### 3.1 Behavioural data

Hits, false alarms, recognition accuracy, and response bias are detailed in Supplementary Fig. 1 and Supplementary Table 2. Compared with the controls, the recognition accuracy for scenes and words was lower in both lmTLE patients (scenes: controls: *Mdn* = 83.3, *Min* = 31.9, *Max* = 96.5, lmTLE: *Mdn* = 66.0, *Min* = 21.5, *Max* = 92.4, *U* = 444.0, *p* = 0.01, *d* = 0.74; words: controls: *Mdn* = 58.3, *Min* = 29.2, *Max* = 83.3, lmTLE: *Mdn* = 29.2, *Min* = 8.3, *Max* = 86.8, *U* = 515.0, *p* = 0.0004, *d* = 1.12) and rmTLE patients (scenes: rmTLE: *Mdn* = 55.2, *Min* = 16.7, *Max* = 93.1, *U* = 403.5, *p* = 0.0006, *d* = 1.20; words: rmTLE: *Mdn* = 42.4, *Min* = 0, *Max* = 72.9, *U* = 370.0, *p* = 0.008, *d* = 0.87). For faces, rmTLE patients performed worse than controls (controls: *Md* = 27.1, *Min* = -4.9, *Max* = 48.6, rmTLE: *Mdn* = 14.9, *Min* = -4.9, *Max* = 32.6, *U* = 358.0, *p* = 0.02, *d* = 0.77) and in tendency also worse than lmTLE patients (lmTLE: *Mdn* = 21.9, *Min* = -6.3, *Max* = 52.8, *U* = 460.5, *p* = 0.08, *d* = 0.49).

### 3.2 Blocked fMRI analysis: Encoding scenes, faces, and words

**Fig. 1:**
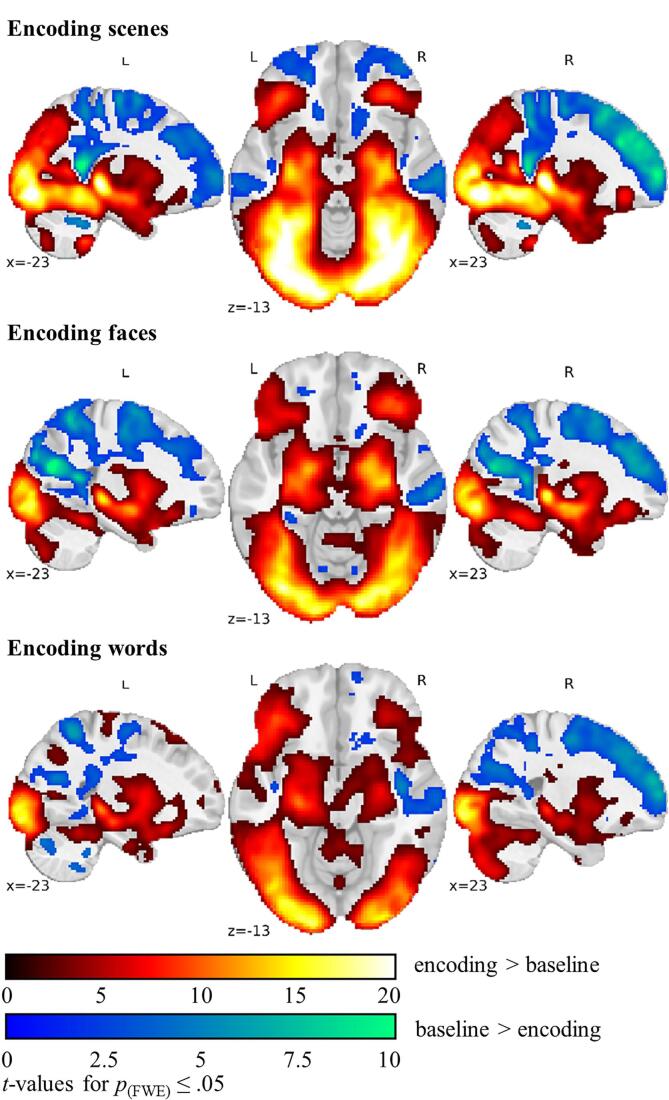
**fMRI activation encoding vs. baseline.** Across-group fMRI activation of encoding scenes, faces and words displayed in MNI-152 space. Colour code indicates *t*-values significant at *p*_(perm.)_ ≤ 0.05 FWE-corrected. Abbreviations: fMRI, functional magnetic resonance imaging; FWE, family-wise error rate.

As shown in Fig. 1, across groups the blocked fMRI analyses of encoding scenes, faces, and words revealed for each of the three conditions widespread bilateral anterior and posterior mesio-temporal, lateral temporal, frontal, basal ganglia, parietal, and occipital activation. In detail, frontal regions included parts of the frontal and central opercular cortex, the anterior cingulate, paracingulate, precentral and inferior, middle and superior frontal gyrus, the insula, the frontal orbital cortex, and the frontal pole.

#### 3.2.1 mTLE patients versus controls

**Fig. 2:**
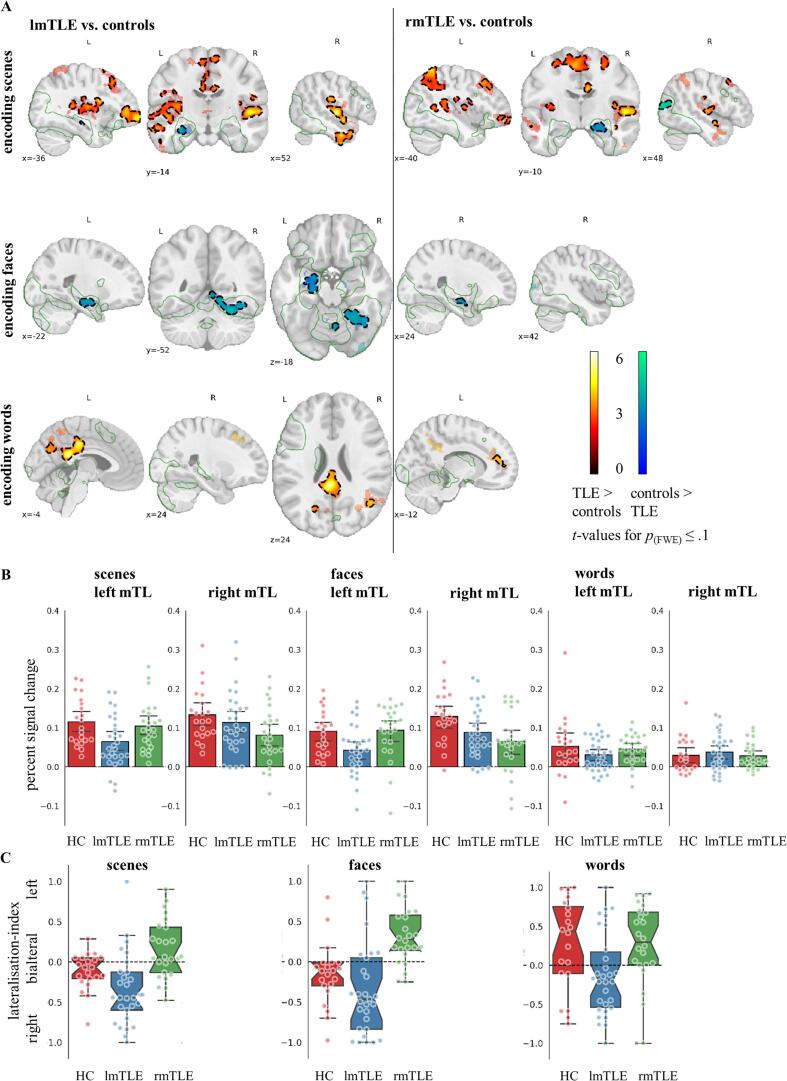
**Group comparisons of fMRI activation encoding versus baseline**. **A** fMRI activation of encoding scenes, faces, and words comparing lmTLE and rmTLE patients with controls. The colour code indicates *t*-values significant at *p*_(perm.)_ ≤ 0.1 FWE-corrected with small volume correction in the mTL. The non-transparent colour framed by a black dotted line indicates *p*_(perm.)_ ≤ 0.05 FWE-corrected. The green outline shows where controls had greater activation during encoding than at baseline. Data are displayed in MNI-152 space. **B** Bar plots with overlaid swarm plots, showing mean percent signal change of the encoding activation (compared to baseline) of controls and lmTLE and rmTLE patients in the mTL. The anatomically defined mTL ROI comprised the hippocampus and amygdala. Whiskers indicate 95% confidence intervals. **C** Lateralisation-indices: Box plot diagrams with overlaid swarm plots, showing the median fMRI lateralisation in the mTL for scenes, faces, and words in controls and lmTLE and rmTLE patients as well as values of individual participants. Notches indicate 95% confidence intervals. Note that in two participants the scene and in three participants each the face and word lateralisation-index could not be calculated, because both mTL sides did not exceed the lateralisation-index-forming voxel threshold of *z* >1.96. Abbreviations: fMRI, functional magnetic resonance imaging; HC, healthy controls; lmTLE, left mesial temporal lobe epilepsy; rmTLE, right mesial temporal lobe epilepsy; mTL, mesial temporal lobe; ROI, region of interest. (For interpretation of the references to colour in this figure legend, the reader is referred to the web version of this article.)

As shown in the top row of Fig. 2A and detailed in Table 2, there were significant group differences for encoding scenes between lmTLE and rmTLE patients and controls. Both patient groups displayed reduced activation in the anterior hippocampus and the amygdala of the epileptogenic hemisphere. Further, rmTLE patients had reduced right lateral occipital activation. lmTLE patients exhibited increased activation bilaterally in predominantly anterior temporo-lateral regions and in left basal ganglia, insula, and frontal regions including the frontal pole, the frontal orbital cortex and the precentral, superior frontal and middle frontal gyrus. At a threshold of *p*_(FWE)_ ≤ 0.1 activation was bilateral in the frontal pole and basal ganglia and additionally encompassed the left inferior frontal gyrus. rmTLE patients demonstrated increased activation in the left basal ganglia, postcentral gyrus, superior parietal lobule, middle frontal gyrus and frontal pole, in right anterior temporo-lateral regions, and bilateral insula. These were more widespread and additionally encompassed the left frontal orbital cortex, superior and inferior frontal gyrus and anterior temporo-lateral and temporo-occpital region at a threshold of *p*_(FWE)_ ≤ 0.1. lmTLE and rmTLE patients also exhibited clusters of more activation in regions, where the overall contrast showed less activation during encoding than during baseline, amounting to less deactivation in patients. These were mostly in regions associated with the default mode and auditory network including the anterior and posterior cingulate gyrus, the precuneus, the angular gyrus, the frontal pole, the posterior superior temporal gyrus, the planum polare and temporale and the Heschl’s gyrus.

**Table 2:** Group comparisons of fMRI activation during scene encoding. Anatomical labels are based on the Harvard-Oxford atlas.

Hemisphere		Peak	Cluster mm^3^ [%]a)	*t*	Peak	Cluster mm^3^ [%]a)	*t*
Encoding scenes							
HC >		lmTLE patients			rmTLE patients		
Left	amygdalab)	−25 −5 −21	2368 [38]	3.0*			
	hippocampusb)	−33 −15 −18	2368 [62]	4.0*			
Right	amygdalab)				22 −7 −15	2384 [47]	3.9**
	hippocampusb)				22 −23 −17	2384 [53]	3.2*
	lateral occipital cortex				48 −81 12	3000 [99]	5.2*

HC <							
Left	central opercular cortex	−56 −23 21	45,960 [9]	3.7*	−44 −6 10	99,312 [3]	3.6*
	cingulate gyrus anterior division	−12 44 10	69,128 [2]	3.0*		99,312 [<1]	
					−11 44 8	35,384 [2]	2.5
	inferior frontal gyrus pars triangularis	−54 33 −5	69,128 [1]	2.9	−52 34 −7	35,384 [<1]	2.8
	inferior frontal gyrus pars opercularis	−51 14 8	496 [1 0 0]	2.8	−51 13 9	99,312 [<1]	2.6*
	insula	−36 −13 10	45,960 [11]	3.6*	−35 −19 6	99,312 [4]	4.0*
	middle frontal gyrus	−47 27 32	69,128 [11]	4.0*		99,312 [<1]	
					−45 22 44	8000 [97]	3.7*
	frontal operculum cortex		45,960 [<1]				
	frontal orbital cortex		69,128 [<1]			35,384 [<1]	
	frontal pole	−36 60 −7	69,128 [27]	4.6*	−35 60 −9	35,384 [39]	4.1*
	juxtapositional lobule cortex	−7 −13 62	69,128 [1]	3.3*	−7 −11 57	99,312 [2]	3.9*
	paracingulate gyrus	−11 40 18	69,128 [6]	3.7*	−7 34 26	35,384 [9]	2.7
	precentral gyrus	−1 −17 48	69,128 [3]	2.9*	−21 −17 60	99,312 [7]	3.9*
		−57 2 10	45,960 [1]	2.5	−11 −23 72	320 [1 0 0]	3.0
			14,352 [3]				
	superior frontal gyrus	−27 12 66	69,128 [11]	4.5*	−27 11 64	99,312 [3]	3.4
					−15 30 56	35,384 [9]	3.5*
					−19 27 40	8000 [2]	2.1
	heschl's gyrus		45,960 [5]		−49 −21 1	99,312 [1]	3.0*
	inferior temporal gyrus		45,960 [<1]			1184 [14]	
	middle temporal gyrus	−57 −7 −31	45,960 [20]	4.3*	−47 −57 10	99,312 [3]	3.7*
					−59 −59 −7	1184 [76]	3.3
	planum polare	−47 −19 −3	45,960 [3]	5.0**	−51 −8 −5	99,312 [<1]	2.3
	planum temporale	−53 −31 9	45,960 [10]	4.5**	−59 −30 10	99,312 [4]	3.9*
	superior temporal gyrus	−55 3 −9	45,960 [9]	4.1*	−53 −7 −11	99,312 [2]	2.3
	temporal pole		45,960 [2]				
		−55 10 −27	352 [1 0 0]	3.8			
	angular gyrus		45,960 [<1]		−48 −59 48	99,312 [5]	3.6*
		−47 −59 20	14,352 [23]	3.7*			
	cingulate gyrus posterior division	−3 −27 42	69,128 [11]	3.2*	−3 −24 44	99,312 [6]	3.4*
	parietal operculum cortex	−59 −37 26	45,960 [5]	2.5		99,312 [1]	
	postcentral gyrus		45,960 [2]		−22 −45 72	99,312 [4]	4.3*
		−22 −43 72	14,352 [21]	4.1*			
	precuneus cortex	−11 −65 34	69,128 [6]	3.7*	−8 −51 60	99,312 [<1]	2.4*
					−11 −67 46	1568 [99]	4.4
	superior parietal lobule	−31 −45 60	14,352 [21]	4.0*	−43 −53 58	99,312 [4]	3.9*
	supramarginal gyrus	−64 −43 28	45,960 [2]	2.7	−57 −31 48	99,312 [7]	3.2*
			14,352 [3]				
	caudate		45,960 [<1]				
	pallidum	−23 −5 −2	45,960 [3]	4.1*		99,312 [<1]	
	putamen	−25 −6 14	45,960 [5]	2.6*	−27 −5 10	99,312 [4]	2.7*
	thalamus	−7 −7 2	45,960 [3]	4.9*			
	lateral occipital cortex	−42 −61 56	14,352 [29]	3.2*	−43 −60 58	99,312 [4]	3.9*
						1184 [11]	
Right	central opercular cortex		24,488 [9]			16,960 [16]	
	cingulate gyrus anterior division	8 4 38	69,128 [2]	2.9		99,312 [<1]	
						35,384 [3]	
	insula	40 −11 10	24,488 [5]	3.3*	42 −9 9	16,960 [11]	3.3*
	middle frontal gyrus					99,312 [<1]	
					38 20 43	35,384 [18]	4.0*
	frontal pole	10 60 28	69,128 [2]	3.3	22 38 34	35,384 [6]	3.6*
					18 60 24	840 [1 0 0]	3.2
	juxtapositional lobule cortex	8 −5 52	69,128 [2]	2.9	7 −7 54	99,312 [2]	3.4*
	paracingulate gyrus	4 47 18	69,128 [2]	2.6*	10 32 36	35,384 [10]	3.3*
	precentral gyrus	1 −21 49	69,128 [3]	3.0*	8 −19 64	99,312 [9]	3.8*
			24,488 [2]				
			2280 [9]				
	superior frontal gyrus		69,128 [<1]		24 18 54	99,312 [2]	3.5
						35,384 [1]	
	heschl's gyrus	53 −11 2	24,488 [9]	5.4**	54 −11 2	16,960 [11]	4.7*
	inferior temporal gyrus	51 −5 −33	24,488 [11]	4.2*	50 −3 −35	16,960 [3]	3.2
	middle temporal gyrus	44 −29 −5	24,488 [5]	2.9	62 −25 −5	16,960 [4]	3.5*
	parahippocampal gyrus anterior division	24 −9 −39	24,488 [1]	3.8*			
	planum polare		24,488 [9]		54 2 −7	16,960 [13]	4.4*
	planum temporale	57 −21 8	24,488 [10]	3.7*		16,960 [10]	
	superior temporal gyrus		24,488 [13]		70 −27 4	16,960 [17]	5.2*
	temporal fusiform cortex	38 −5 −41	24,488 [7]	3.8*			
	temporal pole	52 10 −31	24,488 [8]	3.6*	50 10 −29	16,960 [4]	3.2
	angular gyrus				47 −50 52	3280 [40]	3.0
	cingulate gyrus posterior division		69,128 [6]		6 −25 26	99,312 [6]	4.9**
	parietal operculum cortex	53 −21 22	24,488 [7]	3.9*	55 −21 22	16,960 [8]	3.4*
	postcentral gyrus	56 −7 23	24,488 [2]	2.9	16 −37 71	99,312 [5]	3.8*
		16 −37 70	2280 [79]	3.3		16,960 [<1]	
	precuneus cortex				12 −69 40	99,312 [4]	3.9*
	superior parietal lobule	26 −41 62	2280 [11]	3.1	24 −43 69	99,312 [1]	3.0*
						3280 [4]	
	supramarginal gyrus		24,488 [<1]		50 −45 44	3280 [15]	3.2
	pallidum	26 −5 −3	45,960 [1]	3.6*			
	putamen		45,960 [1]				
			192 [62]				
	thalamus	8 −3 3	45,960 [1]	2.9			
	cuneal cortex					99,312 [<1]	
	lateral occipital cortex				38 −73 46	3280 [40]	3.6

Abbreviations: fMRI, functional magnetic resonance imaging; FWE, family-wise error rate; HC, healthy controls; lmTLE, left mesial temporal lobe epilepsy; rmTLE, right mesial temporal lobe epilepsy.

a) Cluster size (in mm^3^) of significant clusters at *p*_(FWE)_ ≤ 0.1 revealed by group comparison [% of cluster in the respective region].

b) Small volume correction in the mesial temporal lobe.

* *p*_(FWE)_ ≤ 0.05

** *p*_(FWE)_ ≤ 0.01.

For face encoding, lmTLE and rmTLE patients had less activity in the epileptogenic mTL encompassing the anterior hippocampus and amygdala. In addition, lmTLE patients exhibited reduced activation in the right occipital fusiform, lateral occipital and lingual gyrus. These effects are visualised in Fig. 2A (middle row) and detailed in Table 3.

**Table 3:** Group comparisons of fMRI activation during face and word encoding. Anatomical labels are based on the Harvard-Oxford atlas.

Hemisphere	Peak	Cluster mm^3^ [%]a)	*t*	Peak	Cluster mm^3^ [%]a)	*t*	
Encoding Faces							
HC >	lmTLE patients		rmTLE patients				
Left	amygdalab)	−23 −7 −17	3496 [37]	3.8**			
	hippocampusb)	−25 −16 −14	3496 [63]	3.7**			
Right	amygdala					576 [38]	
	amygdalab)				14 −10 −15	2760 [59]	5.8**
	hippocampus					576 [39]	
	hippocampusb)	14 −13 −19	136 [88]	3.8	16 −15 −17	2760 [40]	5.1**
	temporal occipital fusiform cortex	40 −59 −15	12,512 [26]	4.7*			
	lateral occipital cortex		12,512 [2]		42 −87 8	528 [73]	3.1
		38 −87 8	2464 [46]	2.9*			
		34 −89 −19	712 [80]	4.6			
	lingual gyrus	14 −53 −11	12,512 [17]	4.1*			
	occipital fusiform gyrus	32 −70 −11	12,512 [15]	4.4*			
	occipital pole	30 −92 12	2464 [54]	4.1*			
			712 [20]				

Encoding Words							

HC <	lmTLE patients		rmTLE patients				

Left	cingulate gyrus anterior division					6336 [16]	
	frontal pole				−3 64 10	6336 [16]	3.7
	paracingulate gyrus		248 [87]		−5 56 10	6336 [61]	4.0*
	precentral gyrus		20,872 [<1]				
	cingulate gyrus posterior division	−4 −41 22	20,872 [38]	5.8**	−13 −46 32	2184 [55]	4.0
					−5 −25 38	120 [1 0 0]	4.2
	precuneus cortex	−7 −69 30	20,872 [25]	3.9*	−11 −63 50	2184 [36]	4.2
			1184 [72]				
	cuneal cortex		20,872 [2]				
Right	cingulate gyrus anterior division					176 [95]	
	superior frontal gyrus	22 32 44	1040 [92]	4.4			
	middle temporal gyrus		5272 [10]				
	angular gyrus		5272 [36]				
	cingulate gyrus posterior division	6 −35 32	20,872 [24]	4.1**		2184 [7]	
	precuneus cortex	16 −67 30	20,872 [9]	4.1			
			1184 [27]				
	lateral occipital cortex	45 −61 23	5272 [53]	4.0*			
			312 [95]				

Abbreviations: fMRI, functional magnetic resonance imaging; FWE, family-wise error rate; HC, healthy controls; lmTLE, left mesial temporal lobe epilepsy; rmTLE, right mesial temporal lobe epilepsy.

a) Cluster size (in mm^3^) of significant clusters at *p*_(FWE)_ ≤ 0.1 revealed by group comparison [% of cluster in the respective region].

b) Small volume correction in the mesial temporal lobe.

* *p*_(FWE)_ ≤ 0.05.

** *p*_(FWE)_ ≤ 0.01.

The bottom row of Fig. 2A shows that for word encoding, rmTLE patients exhibited increased activation in the left anterior cingulate and paracingulate gyrus. lmTLE patients had increased activation in the right lateral occipital cortex at a threshold of *p*_(FWE)_ ≤ 0.1. Further, lmTLE patients showed significantly decreased deactivation in similar regions as during scene encoding, namely the posterior cingulate and posterior middle temporal gyrus, and the angular and precuneus cortex. At a lower threshold of *p*_(FWE)_ ≤ 0.1 this held also for rmTLE patients having decreased deactivation in the posterior cingulate gyrus and precuneus cortex. The extent to which individual regions were differently activated is further described in Table 3.

Lateralisation-indices for mTL activity are depicted in Fig. 2C. In HCs, they indicate slight right lateralisation for scenes and faces, and strong left lateralisation for words. In lmTLE patients, the distribution of individual lateralisation is shifted towards the right mTL during scene encoding. Likewise, there is a shift to the right for most lmTLE patients during face encoding, although there are several outliers. For word encoding, most lmTLE patients show right mTLE lateralisation, but the range is wide and several maintain left lateralisation. By contrast, the distribution of rmTLE patients’ lateralisation is shifted relatively homogeneously towards the left mTL during scene, face, and word encoding.

### 3.3 fMRI event-related analyses: Subsequent memory effect of scenes, faces and words

**Fig. 3:**
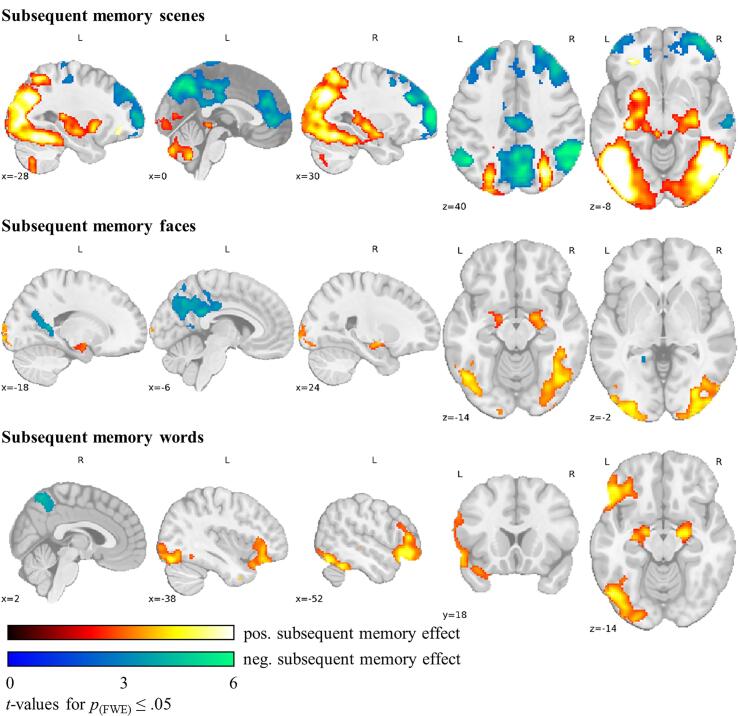
**Across-group fMRI activation of the subsequent memory effect**. Across-group fMRI activation of the positive and negative subsequent memory effect for scenes, faces, and words. The colour code indicates *t*-values significant at *p*_(perm.)_ ≤ 0.05 FWE-corrected with small volume correction in the mTL. Activation is displayed in MNI-152 space. Abbreviations: fMRI, functional magnetic resonance imaging; FWE, family-wise error rate.

Fig. 3 illustrates the subsequent memory effect for the different stimulus classes. Across groups, stronger activation for subsequently remembered compared with subsequently forgotten scenes was apparent in bilateral anterior and posterior mesio-temporal, lateral temporal, basal ganglia, parietal, and occipital regions as well as in left inferior, orbital and precentral frontal regions, the insula, and the frontal pole. Subsequently remembered faces exhibited only bilateral anterior mesio-temporal, lateral temporal and occipital activation. For words, the subsequent memory effect was seen in bilateral anterior mesio-temporal and almost exclusively in left-sided lateral temporal and occipital regions as well as widespread in left frontal regions including the inferior, middle, orbital, and precentral frontal gyrus, and the frontal operculum and pole.

There was a negative subsequent memory effect with increased activation for subsequently forgotten compared with remembered items particularly in regions associated with the default mode network. It was widespread for scenes and more circumscribed for faces and words (see Fig. 3).

#### 3.3.1 mTLE patients versus controls

**Fig. 4:**
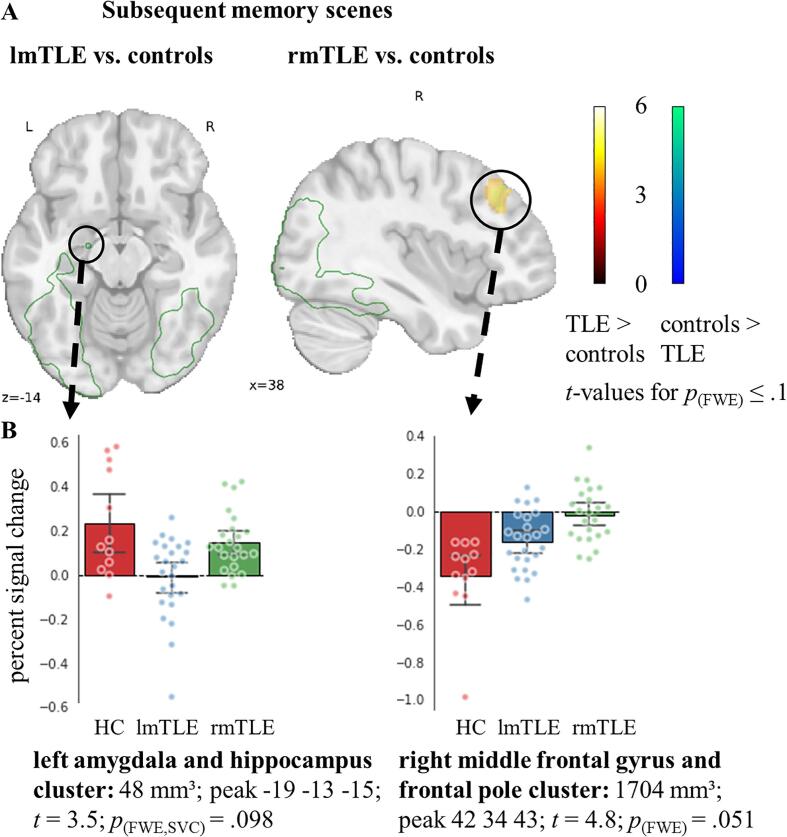
**Group comparisons of fMRI activation of the subsequent memory contrast**. **A** Group differences in the subsequent memory effect of scenes when comparing lmTLE (left) and rmTLE (right) patients with controls. The colour code indicates t-values significant at *p*_(perm.)_ ≤ 0.1 FWE-corrected with small volume correction in the mTL. The green outline indicates subsequent memory activation for controls. Activation is shown in MNI-152 space. There was no significant between group difference in the subsequent memory effect of faces and words. **B** Bar plots with overlaid swarm plots, showing mean percent signal change of subsequent memory activation of controls and lmTLE and rmTLE patients for clusters significant in the group comparisons. Clusters were defined using *p*_(FWE)_ ≤ 0.1 with small volume correction in the mTL. Whiskers indicate 95% confidence intervals. Abbreviations: fMRI, functional magnetic resonance imaging; FWE, family-wise error rate; HC, healthy controls; lmTLE, left mesial temporal lobe epilepsy; rmTLE, right mesial temporal lobe epilepsy. (For interpretation of the references to colour in this figure legend, the reader is referred to the web version of this article.)

Only in the subsequent memory effect of scenes were there marginal group differences (*p*_(FWE)_ ≤ 0.1). lmTLE patients exhibited in a small cluster weaker left amygdala and anterior hippocampus activation for subsequently remembered scenes. rmTLE patients showed increased activation in the right middle frontal gyrus and frontal pole (see Fig. 4A). Note, however, that overall this region was associated with the negative subsequent memory effect (see Fig. 3), implying increased activation for subsequently forgotten scenes. Fig. 4B illustrates the distribution in the individual groups of participants.

### 3.4 Correlation between encoding activation and memory performance in mTLE patients

**Fig. 5:**
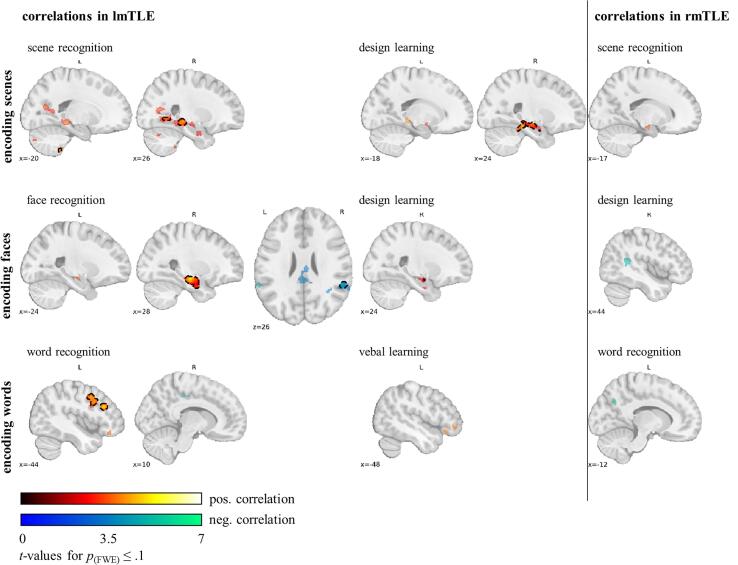
**Correlations between the encoding versus baselin**e **fMRI contrast and memory performance in mTLE patients**. Correlations of fMRI activation of encoding scenes, faces, and words and the respective recognition accuracy and separately determined neuropsychological verbal and design learning scores. The colour code indicates *t*-values significant at *p*_(perm.)_ ≤ 0.1 FWE-corrected with small volume correction in the mTL. The non-transparent colour framed by a black dotted line indicates *p*_(perm.)_ ≤ 0.05 FWE-corrected. Data are displayed in MNI-152 space. Abbreviations: fMRI, functional magnetic resonance imaging; FWE, family-wise error rate; lmTLE, left mesial temporal lobe epilepsy; rmTLE, right mesial temporal lobe epilepsy; mTL, mesial temporal lobe.

We further correlated encoding-related fMRI activation in lmTLE and rmTLE patients with the post-scanner recognition accuracy and verbal and design learning scores to identify functional relevance of activation (see Fig. 5 and Table 4, Table 5). Given that we had to limit the subsequent memory analysis to a subgroup of patients, we decided to correlate memory performance with the general encoding-related activation (learning vs. baseline).

**Table 4:** Correlations of scene encoding fMRI activation with memory performance in lmTLE and rmTLE patients. Anatomical labels are based on the Harvard-Oxford atlas.

Hemisphere		Peak	Cluster mm^3^ [%]a)	*t*	Peak	Cluster mm^3^ [%]a)	*t*
Encoding Scenes		Scene Recognition			Design learning		
lmTLE patients							
Pos. correlation							
Left	amygdala					12,872 [1]	
	hippocampus	−19 −33 −9	3776 [12]	4.2	−17 −33 −7	392 [39]	5.2
	middle temporal gyrus	−47 −53 6	216 [1 0 0]	4.8			
	parahippocampal gyrus posterior division	−30 −33 −19	3776 [17]	2.6			
	temporal fusiform cortex	−33 −41 −19	3776 [21]	3.3			
	temporal occipital fusiform cortex	−39 −51 −17	3776 [28]	3.1			
	cingulate gyrus posterior division	−7 −47 2	19,640 [2]	3.5			
	precuneus cortex	−17 −57 10	19,640 [8]	3.6			
	pallidum				−15 −3 −7	12,872 [4]	5.2*
	thalamus		3776 [7]			12,872 [2]	
						392 [37]	
	cuneal cortex		19,640 [<1]				
	intracalcarine cortex	−1 −71 10	19,640 [6]	3.6			
	lingual gyrus		19,640 [3]				
			3776 [3]				
	occipital fusiform gyrus		3776 [4]				
	supracalcarine cortex	−15 −67 14	19,640 [2]	3.5			

Right	frontal pole				48 36 2	216 [63]	6.4
	amygdala		19,640 [1]		16 −5 −19	12,872 [10]	5.3*
	amygdalab)	26 −13 −13	2064 [43]	2.9	16 −9 −16	3256 [42]	5.0**
	hippocampus	24 −29 −9	19,640 [9]	4.5*	16 −39 6	12,872 [10]	3.5
	hippocampusb)	24 −29 −8	2064 [56]	4.5**	20 −29 −9	3256 [57]	3.8*
		19 −39 4	160 [1 0 0]	3.3			
	inferior temporal gyrus		1504 [32]				
	middle temporal gyrus	58 −47 −9	1504 [52]	3.7			
	parahippocampal gyrus posterior division		19,640 [4]		22 −33 −15	12,872 [13]	4.7*
	temporal fusiform cortex				30 −29 −25	12,872 [3]	3.9
	temporal occipital fusiform cortex	32 −54 −7	19,640 [6]	3.6*		12,872 [1]	
	cingulate gyrus posterior division	15 −47 6	19,640 [7]	4.8*		12,872 [2]	
	precuneus cortex	18 −61 16	19,640 [7]	4.0*			
	pallidum					12,872 [3]	
	putamen		19,640 [<1]				
	thalamus		19,640 [1]		8 −34 8	12,872 [5]	3.4
	intracalcarine cortex	15 −75 4	19,640 [7]	3.0			
	lateral occipital cortex	52 −61 −1	1504 [16]	3.5			
	lingual gyrus	12 −43 −3	19,640 [23]	4.4*			
	occipital fusiform gyrus		19,640 [2]				
	supracalcarine cortex		19,640 [3]				

rmTLE patients							

Pos. correlation							
Left	hippocampusb)	−13 −12 −19	224 [68]	4.3*			

Abbreviations: fMRI, functional magnetic resonance imaging; FWE, family-wise error rate; HC, healthy controls; lmTLE, left mesial temporal lobe epilepsy; rmTLE, right mesial temporal lobe epilepsy.

a) Cluster size (in mm^3^) of significant clusters at *p*_(FWE)_ ≤ 0.1 revealed by group comparison [% of cluster in the respective region].

b) Small volume correction in the mesial temporal lobe.

* *p*_(FWE)_ ≤ 0.05.

** *p*_(FWE)_ ≤ 0.01.

**Table 5:** Correlations of face and word encoding fMRI activation with memory performance in lmTLE and rmTLE patients. Anatomical labels are based on the Harvard-Oxford atlas.

Hemisphere		Peak	Cluster mm^3^ [%]a)	*t*	Peak	Cluster mm^3^ [%]a)	*t*
Encoding faces		Face recognition			Design learning		
lmTLE patients							
Pos. correlation							
Left	amygdalab)	−23 −15 −13	208 [54]	4.0			
	hippocampus		208 [46]				
Right	amygdala		1728 [29]				
	amygdalab)	17 −6 −13	5368 [31]	4.1**	19 −6 −17	1832 [58]	3.7*
	hippocampus	26 −17 −13	1728 [67]	5.3*			
	hippocampusb)	24 −17 −15	5368 [69]	5.1**	30 −9 −18	1832 [42]	2.6

Neg. correlation							
Left	precentral gyrus		17,296 [4]				
	cingulate gyrus posterior division	−10 −33 43	17,296 [7]	5.4*			
		−3 −37 26	1272 [48]	3.7			
	postcentral gyrus	−5 −45 72	17,296 [2]	3.3			
	precuneus cortex	−11 −73 46	17,296 [30]	4.7*			
	supramarginal gyrus	−64 −45 22	392 [1 0 0]	6.3*			
	lateral occipital cortex	−11 −85 48	17,296 [11]	3.9*			
Right	frontal pole	42 58 12	232 [1 0 0]	4.4			
	precentral gyrus		17,296 [3]				
	middle temporal gyrus	57 −23 −7	816 [71]	4.6			
	superior temporal gyrus	60 −23 −3	816 [26]	4.5			
	angular gyrus	50 −53 46	7632 [58]	5.0*			
	cingulate gyrus posterior division	10 −29 42	17,296 [11]	3.9*			
		6 −19 28	1272 [51]	3.5			
	postcentral gyrus		17,296 [2]				
	precuneus cortex	10 −69 52	17,296 [25]	5.0*			
	supramarginal gyrus	58 −44 49	7632 [39]	5.5**			
	lateral occipital cortex	12 −67 54	17,296 [3]	4.9*			
			7632 [3]				

rmTLE patients							
Neg. correlation							
Right	middle temporal gyrus					768 [40]	
	angular gyrus					768 [24]	
	supramarginal gyrus					768 [35]	

Encoding words		Word recognition			Verbal learning		
lmTLE patients							
Pos. correlation							
Left	inferior frontal gyrus pars triangularis		8888 [7]				
		−51 34 4	1112 [55]	4.1			
	inferior frontal gyrus pars opercularis	−56 20 22	8888 [21]	6.3**			
	middle frontal gyrus	−51 27 26	8888 [58]	6.5**			
		−32 16 50	2792 [56]	4.3			
	frontal orbital cortex		280 [43]			416 [85]	
	frontal pole	−53 42 −9	1112 [42]	3.7	−47 42 −6	264 [1 0 0]	4.0
		−43 38 −17	280 [57]	4.1			
	precentral gyrus		8888 [13]				
	superior frontal gyrus	−19 12 68	2792 [43]	5.4*			

Neg. correlation							
Left	precuneus cortex						
Right	cingulate gyrus posterior division	8 −27 44	120 [87]	5.0			

rmTLE patients							
Neg. correlation							
Left	precuneus cortex	−13 −65 34	224 [1 0 0]	6.0			

Abbreviations: fMRI, functional magnetic resonance imaging; FWE, family-wise error rate; HC, healthy controls; lmTLE, left mesial temporal lobe epilepsy; rmTLE, right mesial temporal lobe epilepsy.

a) Cluster size (in mm^3^) of significant clusters at *p*_(FWE)_ ≤ 0.1 revealed by group comparison [% of cluster in the respective region].

b) Small volume correction in the mesial temporal lobe.

* *p*_(FWE)_ ≤ 0.05.

** *p*_(FWE)_ ≤ 0.01.

For scene encoding and lmTLE patients, increased activation in the right posterior hippocampus, parahippocampal gyrus, fusiform cortex, posterior cingulate gyrus, precuneus, lingual gyrus and supracalcarine cortex were associated with better scene recognition accuracy. There was a similar pattern in the left hemisphere at a lower threshold of *p*_(FWE)_ ≤ 0.1. Comparably increased activation in the right amygdala, the anterior and posterior hippocampus, the parahippocampal gyrus and bilateral pallidum was associated with better design learning scores. Again, this held in tendency also for the left posterior hippocampus, and the right fusiform cortex and frontal pole (*p*_(FWE)_ ≤ 0.1). In rmTLE patients there was an association of increased left anterior hippocampus activation and better scene recognition accuracy.

For face encoding and lmTLE patients, increased activation in the right amygdala and anterior hippocampus were associated with both better face recognition accuracy and design learning scores. At a threshold of *p*_(FWE, SVC)_ ≤ 0.1 a more circumscribed activation cluster in the left amygdala and anterior hippocampus also correlated with better face recognition accuracy. Further, there were negative correlations between encoding activation and face recognition accuracy in the pre- and postcentral gyrus, the supramarginal, angular and posterior cingulate gyrus, the precuneus and lateral occipital cortex. rmTLE patients showed, only at a lower threshold of *p*_(FWE, SVC)_ ≤ 0.1, similar negative correlations between encoding activation and design learning scores in the supramarginal, angular and posterior middle temporal gyrus.

For word encoding and lmTLE patients, increased activation in the left inferior frontal, middle frontal and precentral gyrus was associated with better word recognition accuracy. At a lower threshold of *p*_(FWE, SVC)_ ≤ 0.1 this held also for the left frontal orbital cortex and frontal pole for both word recognition accuracy and verbal learning scores. Correlations of right amygdala and anterior hippocampus activation and word recognition were only found at an even lower threshold of *p*_(FWE, SVC)_ ≤ 0.15 (peak: 24, −7, −19, *t* = 3.0, *p*_(FWE, SVC)_ = 0.12). Further, in lmTLE and rmTLE patients there was a tendency for negative correlations between activation in the precuneus and posterior cingulate gyrus and recognition accuracy (*p*_(FWE)_ ≤ 0.1).

### 3.5 Impact of clinical characteristics on memory performance in mTLE patients

To identify potential clinical features which impact memory performance, we calculated spearman correlations of the recognition accuracy of each condition, and verbal and design learning scores with epilepsy onset, epilepsy duration, and AED load. In lmTLE patients higher drug load was associated with lower memory performances (scene recognition accuracy *r* = −0.50, *p* = 0.005, design learning *r* = −0.34, *p* = 0.07). Further, memory performance negatively correlated with epilepsy duration (scene recognition accuracy *r* = −0.41, *p* = 0.03, design learning *r* = −0.32, *p* = 0.10) and also somewhat with epilepsy onset (word recognition accuracy *r* = −0.32, *p* = 0.09, verbal learning *r* = −0.30, *p* = 0.11). In rmTLE patients higher drug load was also associated with lower memory performance (design learning *r* = −0.39, *p* = 0.05). All other comparison revealed *ps* > 0.15.

## 4 Discussion

We studied whole-brain neural correlates of memory encoding and subsequent memory in mTLE patients and controls. The current fMRI paradigm of encoding scenes, faces, and words proved well-suited to measure encoding as well as subsequent memory-related activation within and outside of the mTL. Scenes elicited especially strong and bilateral, if slightly right dominant, encoding-related haemodynamic activation and large subsequent memory effects, providing a sound basis for detecting disease-related alterations. Analysis of encoding-related haemodynamic activity revealed decreased activation in the epileptogenic mTL and extra-temporal increases in mTLE patients. However, subsequent memory analyses provided only marginal systematic group differences specifically related to memory formation. Correlations of encoding-related activation and memory performance revealed a predominant role of the mTL and, solely for words and lmTLE patients, of left frontal regions. Especially correlations between activation in the contralateral mTL and behaviour may reflect disease-induced reorganisation within the mTL system.

### 4.1 Behavioural results

Compared with controls, both rmTLE and lmTLE patients had reduced recognition accuracies for scenes and words, while only rmTLE patients showed decreased face recognition accuracy. Direct comparison of lmTLE and rmTLE patients revealed a tendency for worse face recognition accuracy in rmTLE patients.

The finding of reduced word recognition irrespective of the side of mTLE does not support theories of a predominant role of the left mTL in verbal memory. (Jeyaraj et al., 2013, Willment and Golby, 2013) Results are instead in line with Sailing (Saling, 2009), who casts doubt on strictly dichotomous material-specific lateralisation in mTLE. This is further supported by a number of previous imaging, (Dupont et al., 2002, Maccotta et al., 2007, Sidhu et al., 2013) and behavioural, (Chang et al., 2019, Postma et al., 2020, Reyes et al., 2019, Sidhu et al., 2015b) studies reporting impaired verbal memory in both lTLE and rTLE patients. Further, our task may have implicitly elicited semantic clustering during encoding, which has been found to be equally impaired in lmTLE and rmTLE patients, (Grewe et al., 2020) potentially contributing to rmTLE patients’ verbal memory impairment. Conversely, increased activation in the right mTL might reflect reliance on imaginability and dual-coding strategies during word encoding. (Maguire et al., 2003) Pictorial memory might be reduced in both patient groups because it relies on memory for spatial detail, theoretically more strongly linked to the right hippocampus, and memory for self-generated verbal labels that may depend on the left hippocampus. Due to their structural similarity, faces may be hard to verbalise, causing the more pronounced face-memory impairment in rmTLE.

### 4.2 Neural correlates of encoding

Across-group encoding activation (compared with baseline) was reflected in widespread mesial and lateral temporal, frontal, basal ganglia, and occipital regions and most pronounced for scenes. Deactivation occurred mostly in regions typically associated with the default mode and auditory network. Lateralisation-indices show that mTL activation in HC was slightly right-lateralised for scenes and faces, and strongly left-lateralised for words. In mTLE patients, lateralisation was, on average, shifted towards the mTL contralateral to the epileptogenic focus, a finding that is consistent with previous studies. (e.g. Detre et al., 1998, Towgood et al., 2015)

This general pattern was further reflected in the strongly decreased activation in the contralateral mTL during non-verbal encoding. This might be expected due to the pathology and broadly replicates earlier findings. (Powell et al., 2007, Sidhu et al., 2013, Avila et al., 2006, Cheung et al., 2006, Jokeit et al., 2001) During verbal encoding, the signal change in the mTL was much lower in all groups, probably yielding insufficient power to detect group differences except in lateralisation-indices. lmTLE patients further demonstrated reduced activation for faces in the right occipital fusiform and lingual gyrus. rmTLE patients had less activation for scenes and in tendency also for faces in right lateral occipital regions. These findings agree with theories assuming functional coupling between medial temporal and visual processing regions during stimulus encoding (Ranganath et al., 2005) and demonstrate pathology-specific activation deficits in this system.

Most consistently across conditions and most prominent during scene encoding, mTLE patients exhibited less deactivation than controls in brain regions known to be part of the default mode network, (Raichle, 2015) such as the posterior cingulate gyrus and the precuneus. Further, patients had less deactivation in regions of the auditory network, which has also been established during the resting state. (Raichle, 2015) This region again revealed stronger baseline than task activation in all groups. Sidhu et al. (Sidhu et al., 2013) reported activation changes for mTLE patients in a comparable region. Hill et al. (Hill et al., 2020) and Stevens et al. (Stevens et al., 2008) found activation in this region to be negatively correlated with memory performance and proposed that failure to suppress scanner noise hinders memory formation, a phenomenon that would fit well with our data. We further detected increased activation in predominantly anterior temporo-lateral regions, the insula, the basal ganglia, anterior cingulate gyrus and in the left frontal gyrus, frontal orbital cortex, and frontal pole during encoding of either scenes or words in mTLE patients, thus reflecting widespread extra-mTL recruitment as previously suggested. (Sidhu et al., 2013, Alessio et al., 2013, Dupont et al., 2000, Dupont et al., 2002, Maccotta et al., 2007, Limotai et al., 2018)

Altogether, these findings suggest that mTLE patients exhibit primarily reduced activation during encoding in lesioned brain regions, which impedes memory formation and may increase subjective task difficulty. Consequently, mTLE patients might either deactivate less areas associated with the baseline condition during the encoding task or, conversely, suppress areas associated with the encoding task less during the baseline condition in order to rehearse more to compensate memory impairments. The increased lateral temporal and frontal activation seen during encoding could reflect more effort, reliance on the semantic system, or strategic control of encoding, (Saling, 2009, Simons and Spiers, 2003, Eichenbaum, 2017) although group-level data suggest it is insufficient to consistently compensate for mTL damage.

### 4.3 Neural correlates of subsequent memory

A critical question we addressed was to what extent patients reorganise to compensate for their pathology by using different brain regions compared with controls for subsequent memory formation. Our analysis revealed no region with increased subsequent memory activation in mTLE patients. In detail, the subsequent memory analysis only revealed that compared with controls, lmTLE patients exhibited in tendency lower left amygdala and anterior hippocampus activation during subsequent memory formation of scenes. Given that lmTLE patients, unlike controls, showed no memory benefit for emotional scenes (negative hit − neutral hit: lmTLE patients: *Mdn* = 2.8, *Min* = -19.4, *Max* = 25.0; controls: *Mdn* = 15.3, *Min* = -8.3, *Max* = 33.3; *U* = 95, *p* = 0.08, *d* = 0.61), the reduced anterior mTL activation might also be a correlate of a memory benefit for negative scenes present in controls but not lmTLE patients. This would appear to be in line with reduced emotional memory in lTLE (Múnera et al., 2015) and the notion that co-activation of an intact amygdala may enhance memory formation. (Strange et al., 2014) rmTLE patients instead only showed increased activation in the right middle frontal gyrus and frontal pole, regions overall associated with the negative subsequent memory effect and thus associated with activation for subsequently forgotten scenes. Overall, at least at the group level, the subsequent memory analysis revealed little evidence of temporal or extra-temporal functional reorganisation in mTLE patients. This could be due to highly individual reorganisation patterns which are therefore not significant at group level testing. Moreover, the subsequent memory contrast might be less sensitive despite being more specific than encoding alone. (Sidhu et al., 2015) The finding that regions in which mTLE patients exhibited more activation than controls during encoding compared with baseline were partly involved with subsequent memory formation, at least at more lenient thresholds, might be indicative thereof.

Given that mTLE patients demonstrated typical impairments in memory performance, it seems plausible that memory formation in most of these patients still largely relies on the same brain regions as in controls. On the other hand, some extra mTL regions with increased activation in the encoding vs. baseline contrast in mTLE patients compared with controls also showed up in the subsequent memory analysis, indicating some extra-temporal functional recruitment, as previously suggested by Sidhu et al. (Sidhu et al., 2013) Two other previous studies instead reported stronger contralateral mTL activation in TLE patients than in controls for subsequently remembered stimuli. (Richardson et al., 2003, Powell et al., 2007) However, unlike our study or the ones by Bonelli et al. (Bonelli et al., 2010) and Hill et al. (Hill et al., 2020), the TLE patients in those studies had preserved memory function. Powell et al. (Powell et al., 2007) further reported an inverse correlation between memory performance and degree of contralateral activation for faces and words, which might also be due to the small group (*n* = 7) of the on average unimpaired patients. Accordingly, Sidhu et al. (Sidhu et al., 2013) demonstrated for a larger sample of mTLE patients a positive correlation between memory performance and subsequent memory activation in the mTL contralateral to seizure focus for words and faces. Thus, contralateral mTL reorganisation of subsequent memory may be restricted to only some TLE patients, potentially those with intact memory performance.

### 4.4 Correlation between encoding activation and memory performance in mTLE patients

The correlation analysis of encoding activation and memory performance demonstrated an association between increased contralateral mTL activation and better memory performance. This finding was consistent across stimulus categories and held both for performance within the experimental paradigm and when correlating with clinical tests, adding ecological validity. As was to be expected, the association was stronger for within paradigm correlations. It was also more prominent in lmTLE patients. Additionally, but only in smaller clusters, there was a tendency for increased ipsilateral mTL activation to correlate with better memory performance in lmTLE patients. mTL correlations were more posterior and extended to temporo-lateral and parietal regions for scenes and located anterior for faces and words, irrespective of the hemisphere. Further, increased word encoding activation in left frontal regions (inferior and middle frontal and precentral gyrus, frontal orbital cortex and frontal pole) correlated with better verbal memory in lmTLE patients. Clinically, better memory performance correlated with lower AED load and in lmTLE patients also with earlier epilepsy onset, and shorter epilepsy duration, suggesting that these clinical factors may affect mTL plasticity and contribute to the extent to which ipsi- or contralateral activation subserves memory formation. Albeit age significantly correlated with epilepsy onset and duration in lmTLE patients we would not assume the correlations with memory performance to be a pure age effect. This is because uniquely in lmTLE patients higher age was broadly associated with lower memory performance, whereas no impact of age on memory performance was found in rmTLE patients or controls. Thus, we would speculate the correlation of epilepsy onset and duration with memory performance to be due to epilepsy-induced increased effects of aging, which might only be evident in lmTLE patients due to the longer epilepsy duration in this group (lmTLE patients *M* = 20.2; rmTLE patients *M* = 14.0). This might also contribute to the finding of stronger correlations in lmTLE patients, suggesting that in patients with longer epilepsy duration functional recruitment of especially the contralateral mTL is more relevant to maintain intact memory. However, given that onset, duration and age are all intercorrelated in this dataset, we cannot determine their individual contribution, although it is plausible that these factors have at least partly unique roles. Further, it should also be noted that the lmTLE group was somewhat larger, yielding more power to detect effects.

Overall, these correlations are in line with previous studies indicating reorganisation to the contralateral mTL being functional in both lTLE, (Richardson et al., 2003, Figueiredo et al., 2008) and rTLE patients(Milian et al., 2015). They further reveal functional ipsilateral mTL activation in some lmTLE patients, which were either anterior or posterior depending on stimulus material. Hence, other than previous studies underlining the role of the posterior ipsilateral hippocampus, (Sidhu et al., 2015a, Sidhu et al., 2013), we found evidence for both the anterior and posterior ipsilateral hippocampus to support memory performance. Further, data support a role of left frontal regions for verbal memory performance, (Sidhu et al., 2015a, Sidhu et al., 2013), at least in lmTLE-patients. The pattern as a whole may reflect reorganisation, compensatory strategies, resilience, or their combination.

### 4.5 Conclusion

In summary, we have extended previous studies by investigating general encoding-related activation as well as subsequent memory activation for scenes, faces, and words in a relatively large and homogenous group of mTLE patients and well-matched HCs, simultaneously assessing both mTL and extra-mTL regions. Encoding-related activity was decreased in epileptogenic mTL and increased in extra-temporal regions, which were partly associated with subsequent memory formation but did not correlate with better memory performance. Further, we found decreased deactivation in regions, which have previously been associated with the default mode and auditory network. The findings suggest that successful memory formation in mTLE patients does not systematically rely on different brain regions compared with controls, although we cannot rule out differences on the individual level. The correlations of encoding activation and memory performance in mTLE patients hint towards reorganisation to the contralateral mTL to maintain intact memory and the importance of left frontal regions particularly for verbal memory performance and lmTLE patients.

Although we investigated a relatively large and homogenous group of mTLE patients, an even larger sample would allow us to further specify the individual and joint role of clinical, demographic, and cognitive variables on fMRI activation and functional outcome. This would facilitate more detailed understanding of distinct reorganisation patterns in subgroups. So far, data underline mTL activation as a bottleneck for memory formation with functional reorganisation occurring primarily within the mTL system and further hint towards increased activation in extra-temporal regions in mTLE patients, whose functionality is not entirely clear.

## Declaration of Competing Interest

The authors declare that they have no known competing financial interests or personal relationships that could have appeared to influence the work reported in this paper.
