# Supplementary material for: Whole-brain functional correlates of memory formation in mesial temporal lobe epilepsy
Source: Neuroimage Clin. 2021 Jun 10;31:102723. doi: 10.1016/j.nicl.2021.102723 (PMC8220377; doi:10.1016/j.nicl.2021.102723)
Supplement: Supplementary data 1 [file mmc1.pdf]

Supplementary Material

Supplementary Material I

Table S1

Summary of studies reporting fMRI activation of subsequent memory formation with respect to group comparison of TLE patients and controls.

Study		Bonelli et al.[1,a)]	Powell et al.[2,a)]	Richardson et al.[3,a)]	Hill et al.[4,a),b)]	Sidhu et al.[5,a),b)]
n		41 lTLE<br>31 rTLE	7 lTLE<br>7 rTLE	24 lTLE	4 bilateral<br>5 lTLE<br>7 rTLE	24 lTLE<br>20 rTLE
Words	lTLE	↓ ipsi. hipp.	↑ contra. parahipp.	↑ contra. parahipp.	↔[c)]	*
	rTLE	↓ contra. hipp.	↑ contra. amygdala + hipp.	-	↔	*
Line Drawings	lTLE	↔	↔	-	-	-
	rTLE	↔	↔	-	-	-
Faces	lTLE	↔	↑ contra. parahipp.	-	-	*
	rTLE	↓ ipsi. hipp. (trend)	↔	-	-	*

Note. [a)] analysis of mTL; [b)] whole-brain analysis; [c)] collapsed group of lTLE and bilateral TLE patients; ↑ increased activation compared to controls; ↓ decreased activation compared to controls; ↔ no significant difference; - not studied; * no statistical group comparison with controls. Abbreviations: contra., contralateral to epileptic focus; hipp., hippocampus; ipsi., ipsilateral to epileptic focus; lTLE, left temporal lobe epilepsy; mTL, mesial temporal lobe; parahipp., parahippocampus; rTLE, right temporal lobe epilepsy.

Supplementary Material II

Hits and false alarms

Memory performance is detailed in Supplementary Fig. 1 and Supplementary Table 2. Compared to the controls, the percentage of hits for scenes and words was lower in both lmTLE (scenes: $U = 464.5$, $p = .004$, $d = 0.88$; words: $U = 440.0$, $p = 0.03$, $d = 0.62$) and rmTLE patients (scenes: $U = 451.0$, $p = 0.000006$, $d = 1.83$; words: $U = 409.5$, $p = .0004$, $d = 1.26$). For faces, this was the case only in rmTLE patients ($U = 347.0$, $p = .03$, $d = 0.68$). Further, the percentage of hits was significantly lower in rmTLE patients than lmTLE patients for faces ($U = 474.5$, $p = .05$, $d = 0.56$) and in tendency for scenes ($U = 467.0$, $p = .06$, $d = 0.52$).

The percentage of false alarms for words tended to be higher in lmTLE patients than in controls ($U = 235.0$, $p = .09$, $d = 0.48$). Regarding the false alarms no other between group differences were found (*p*s > .1).

Response bias

The analysis of the response bias demonstrated that rmTLE patients made more conservative responses than lmTLE patients for words ($U = 496.0$, $p = .04$, $d = 0.59$). No other differences in response bias were found (*p*s > .1).

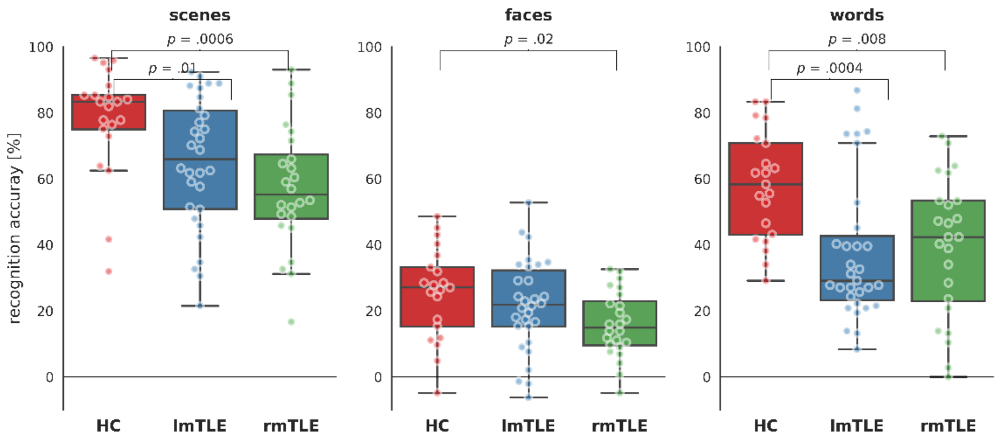

Figure S1. **Recognition accuracy.** Box plot diagrams with overlaid swarm plots, showing the median recognition accuracy (hits – false alarms) for scenes, faces, and words in controls, lmTLE and rmTLE patients as well as within and between-group variability. Abbreviations: HC, healthy controls; lmTLE, left mesial temporal lobe epilepsy; rmTLE, right mesial temporal lobe epilepsy.

Table S2

Percentage of hits and false alarms, recognition accuracy and response bias of scenes, faces and words separately for controls, lmTLE and rmTLE patients.

	%	Controls [*Mdn* (*Min*; *Max*)]	lmTLE patients [*Md*n (*Min*; *Max*)]	rmTLE patients [*Mdn* (*Min*; *Max*)]
Scenes	Hit	87.5 (75.0; 98.6)	77.8 (38.9; 97.2)	65.3 (16.7; 97.2)
	False alarm	2.1 (0; 45.8)	6.3 (0; 35.4)	4.2 (0; 27.1)
	Recognition accuracy	83.3 (31.9; 96.5)	66.0 (21.5; 92.4)	55.2 (16.7; 93.1)
	Response bias	0.35 (0; 0.88)	0.17 (0; 0.69)	0.09 (0; 0.60)
Faces	Hit	54.2 (36.1; 72.2)	57.6 (31.9; 90.3)	46.5 (9.7; 66.7)
	False alarm	35.4 (0; 60.4)	30.2 (6.3; 91.7)	32.3 (0; 56.3)
	Recognition accuracy	27.1 (-4.9; 48.6)	21.9 (-6.3; 52.8)	14.9 (-4.9; 32.6)
	Response bias	0.45 (0; 0.64)	0.41 (0.10; 0.90)	0.41 (0; 0.57)
Words	Hit	77.8 (50.0; 91.7)	70.8 (25.0; 93.1)	61.8 (0; 83.3)
	False alarm	16.7 (2.1; 50.0)	25.0 (2.1; 70.8)	13.5 (0; 60.4)
	Recognition accuracy	58.3 (29.2; 83.3)	29.2 (8.3; 86.8)	42.4 (0; 72.9)
	Response bias	0.42 (0.10; 0.80)	0.47 (0.03; 0.79)	0.27 (0; 0.70)

Note. Abbreviations: lmTLE, left mesial temporal lobe epilepsy; rmTLE, right mesial temporal lobe epilepsy.

Supplementary Material III

Table S3

List of stimuli used in the experiment

pictures	negative	neutral
IAPS pictures[6]	1120	1450
	1205	1675
	1275	1910
	1300	2026
	1304	2038
	1932	2102
	2095	2308
	2205	2372
	2276	2377
	2683	2384
	2688	2390
	2710	2393
	2800	2396
	2811	2397
	2981	2400
	3103	2411
	3181	2435
	3185	2440
	3213	2484
	3230	2487
	3300	2495
	3301	2499
	3350	2513
	3550	2521
	6021	2525
	6311	2575
	6313	2579
	6415	2580
	6520	2594
	6821	2635
	6834	2745
	7380	2749
	8230	2840
	8485	2850
	9000	5471
	9006	5531
	9007	7009
	9040	7187
	9041	7476
	9043	7491
	9140	7512
	9265	7513
	9402	7550
	9421	7632

	9432	8241
	9433	8312
	9480	
	9560	
	9584	
	9611	
	9630	
	9800	
	9810	
	9909	
	9940	
own dataset	child soldier	wooden chair
	burning house	empty dental practice
	mouldy orange	people sitting at a table
	mouldy toast	car
	bloody face (mini-3051)	church building (N77A)
		hiking shoes and socks
		train
		crowned crane
		mallard duck
		pot-bellied pig
		fox
		toad
		bird
		person cooking a meal
faces	negative	neutral
NimStim faces[7]	female	female
	01F_FE_O	01F_NE_C
	02F_FE_O	02F_NE_C
	03F_FE_O	03F_NE_C
	05F_FE_O	05F_NE_C
	06F_FE_O	06F_NE_C
	07F_FE_O	07F_NE_C
	08F_FE_O	08F_NE_C
	09F_FE_O	09F_NE_C
	10F_FE_O	10F_NE_C
	11F_FE_O	11F_NE_C
	17F_FE_O	17F_NE_C
	18F_FE_O	18F_NE_C
	male	male
	20M_FE_O	20M_NE_C
	21M_FE_O	21M_NE_C
	22M_FE_O	22M_NE_C
	23M_FE_O	23M_NE_C
	24M_FE_O	24M_NE_C
	25M_FE_O	25M_NE_C
	27M_FE_O	27M_NE_C
	28M_FE_O	28M_NE_C
	29M_FE_O	29M_NE_C
	30M_FE_O	30M_NE_C

	36M_FE_O	36M_NE_C
	37M_FE_O	37M_NE_C
FACES database[8]	female - young age	female - young age
	010_y_f_f_b	010_y_f_n_a
	020_y_f_f_a	020_y_f_n_a
	022_y_f_f_a	022_y_f_n_a
	040_y_f_f_b	040_y_f_n_a
	048_y_f_f_b	048_y_f_n_a
	054_y_f_f_a	054_y_f_n_a
	098_y_f_f_a	098_y_f_n_a
	101_y_f_f_b	101_y_f_n_a
	106_y_f_f_b	106_y_f_n_a
	115_y_f_f_a	115_y_f_n_a
	163_y_f_f_b	163_y_f_n_a
	182_y_f_f_b	182_y_f_n_a
	male - young age	male - young age
	008_y_m_f_b	008_y_m_n_a
	013_y_m_f_a	013_y_m_n_a
	041_y_m_f_b	041_y_m_n_a
	057_y_m_f_a	057_y_m_n_a
	066_y_m_f_a	066_y_m_n_a
	072_y_m_f_a	072_y_m_n_a
	089_y_m_f_a	089_y_m_n_a
	109_y_m_f_b	109_y_m_n_a
	114_y_m_f_a	114_y_m_n_a
	123_y_m_f_a	123_y_m_n_a
	167_y_m_f_a	167_y_m_n_a
	170_y_m_f_b	170_y_m_n_a
	female - middle age	female - middle age
	006_m_f_f_b	006_m_f_n_a
	011_m_f_f_b	011_m_f_n_b
	019_m_f_f_a	019_m_f_n_b
	029_m_f_f_a	029_m_f_n_a
	035_m_f_f_b	035_m_f_n_a
	050_m_f_f_a	050_m_f_n_b
	052_m_f_f_b	052_m_f_n_a
	061_m_f_f_a	061_m_f_n_a
	064_m_f_f_b	064_m_f_n_b
	073_m_f_f_a	073_m_f_n_b
	080_m_f_f_b	080_m_f_n_a
	084_m_f_f_b	084_m_f_n_b
	male - middle age	male - middle age
	007_m_m_f_a	007_m_m_n_a
	014_m_m_f_a	014_m_m_n_a
	026_m_m_f_a	026_m_m_n_b
	032_m_m_f_b	032_m_m_n_a
	038_m_m_f_b	038_m_m_n_a
	045_m_m_f_a	045_m_m_n_b
	056_m_m_f_a	056_m_m_n_a
	058_m_m_f_b	058_m_m_n_a
	068_m_m_f_b	068_m_m_n_b

	070_m_m_f_a	070_m_m_n_b
	077_m_m_f_b	077_m_m_n_b
	082_m_m_f_a	082_m_m_n_b
	female - old age	female - old age
	005_o_f_f_a	005_o_f_n_b
	012_o_f_f_b	012_o_f_n_b
	021_o_f_f_b	021_o_f_n_a
	024_o_f_f_b	024_o_f_n_a
	036_o_f_f_b	036_o_f_n_a
	044_o_f_f_a	044_o_f_n_a
	047_o_f_f_a	047_o_f_n_a
	055_o_f_f_b	055_o_f_n_b
	060_o_f_f_a	060_o_f_n_a
	067_o_f_f_a	067_o_f_n_b
	075_o_f_f_a	075_o_f_n_a
	079_o_f_f_b	079_o_f_n_b
	male - old age	male - old age
	004_o_m_f_a	004_o_m_n_b
	015_o_m_f_b	015_o_m_n_a
	018_o_m_f_b	018_o_m_n_a
	027_o_m_f_b	027_o_m_n_a
	039_o_m_f_b	039_o_m_n_b
	042_o_m_f_a	042_o_m_n_a
	046_o_m_f_a	046_o_m_n_a
	053_o_m_f_a	053_o_m_n_b
	059_o_m_f_a	059_o_m_n_b
	065_o_m_f_a	065_o_m_n_b
	074_o_m_f_b	074_o_m_n_b
	076_o_m_f_a	076_o_m_n_b
KDEF[9]	female	female
	AF01AFS	AF01NES
	AF06AFS	AF06NES
	AF07AFS	AF07NES
	AF11AFS	AF11NES
	AF13AFS	AF13NES
	AF14AFS	AF14NES
	AF15AFS	AF15NES
	AF16AFS	AF16NES
	AF18AFS	AF18NES
	AF19AFS	AF19NES
	AF30AFS	AF30NES
	AF31AFS	AF31NES
	male	male
	AM01AFS	AM01NES
	AM02AFS	AM02NES
	AM04AFS	AM04NES
	AM05AFS	AM05NES
	AM06AFS	AM06NES
	AM07AFS	AM07NES
	AM08AFS	AM08NES
	AM10AFS	AM10NES

	AM11AFS	AM11NES
	AM13AFS	AM13NES
	AM22AFS	AM22NES
	AM23AFS	AM23NES

words	negative	neutral
own dataset	Alptraum (nightmare)	Aktentasche (briefcase)
	Angst (fear)	Armbeuge (arm bend)
	Beklemmung (anxiety)	Automat (automat)
	Bestie (beast)	Batterie (battery)
	Blamage (disgrace)	Beleg (receipt)
	Blut (blood)	Bewohner (inhabitant)
	Brutalität (brutality)	Biegung (bend)
	Demütigung (humiliation)	Bleistift (pencil)
	Diebstahl (robbery)	Brause (sherbet)
	Diktator (dictator)	Bügeleisen (flat iron)
	Durchfall (diarrhoea)	Computer (computer)
	Eifersucht (jealousy)	Detail (detail)
	Eiter (pus)	Eigenschaft (characteristic)
	Ekel (disgust)	Fahrkarte (ticket)
	Ekzem (eczema)	Faktor (factor)
	Elend (misery)	Flasche (bottle)
	Erpresser (blackmailer)	Flugzeug (aircraft)
	Explosion (explosion)	Gerüst (scaffold)
	Fixer (junkie)	Geschirr (dishes)
	Fluch (curse)	Getreide (grain)
	Folter (torture)	Hausschuhe (slippers)
	Geisel (hostage)	Information (information)
	Geschwür (ulcer)	Kanister (canister)
	Habgier (greed)	Kastanie (chestnut)
	Henker (executioner)	Kasten (box)
	Heroin (heroin)	Kleiderbügel (clothes hanger)
	Hetze (rabble-rousing)	Klingel (bell)
	Hilflosigkeit (helplessness)	Kran (crane)
	Hunger (hunger)	Kurve (curve)
	Hungersnot (famine)	Merkmal (feature)
	Isolation (isolation)	Mikroskop (microscope)
	Kälte (cold)	Motorrad (motorcycle)
	Katastrophe (catastrophe)	Natrium (sodium)
	Kerker (dungeon)	Objekt (object)
	Kreuzigung (crucifixion)	Papier (paper)
	Leiden (suffering)	Partikel (particle)
	Lepra (leprosy)	Plastik (plastic)
	Lügner (liar)	Post (post)
	Lungenkrebs (lung cancer)	Pronomen (pronoun)
	Narbe (scar)	Quadrat (square)
	Nazi (Nazi)	Rasen (lawn)
	Opfer (victim)	Reflex (reflex)
	Panik (panic)	Regal (shelf)
	Perversion (perversion)	Reifen (tire)
	Pisse (piss)	Rolltreppe (escalator)

Rassismus (racism)	Ruder (rudder)
Selbstmord (suicide)	Sicht (view)
Seuche (epidemic)	Siedlung (settlement)
Sklaverei (slavery)	Spiegel (mirror)
Spritze (syringe)	Stellvertretung (representation)
Teufel (devil)	Symbol (symbol)
Tumor (tumor)	Tablett (tray)
Ungerechtigkeit (injustice)	Tastatur (keyboard)
Untergang (doom)	Truhe (chest)
Vergewaltigung (rape)	Turban (turban)
Verrat (betrayal)	Ufer (shore)
Verstümmelung (mutilation)	Unterlage (base)
Wahn (delusion)	Vorhang (curtain)
Warze (wart)	Votum (vote)
Wunde (wound)	Ziegel (brick)

Supplementary References

1. Bonelli SB, Powell HWR, Yogarajah M, *et al.* Imaging memory in temporal lobe epilepsy: predicting the effects of temporal lobe resection. *Brain*. 2010;133(4):1186-1199.
2. Powell HWR, Richardson MP, Symms MR, *et al.* Reorganization of verbal and nonverbal memory in temporal lobe epilepsy due to unilateral hippocampal sclerosis. *Epilepsia*. 2007;48(8):1512-1525.
3. Richardson MP, Strange BA, Duncan JS, Dolan RJ. Preserved verbal memory function in left medial temporal pathology involves reorganisation of function to right medial temporal lobe. *Neuroimage*. 2003;20(Suppl 1):112-119.
4. Hill PF, King DR, Lega BC, Rugg MD. Comparison of fMRI correlates of successful episodic memory encoding in temporal lobe epilepsy patients and healthy controls. *Neuroimage*. 2020;207:116397.
5. Sidhu MK, Stretton J, Winston GP, *et al.* A functional magnetic resonance imaging study mapping the episodic memory encoding network in temporal lobe epilepsy. *Brain*. 2013;136(6):1868-1888.
6. Lang PJ, Bradley MM, Cuthbert BN. International Affective Picture System (IAPS): affective ratings of pictures and instruction manual. *Technical Report A-8*. 2008.
7. Tottenham N, Tanaka JW, Leon AC, *et al.* The NimStim set of facial expressions: judgments from untrained research participants. *Psychiatry Research*. 2009;168(3):242-249.
8. Ebner NC, Riediger M, Lindenberger U. FACES--a database of facial expressions in young, middle-aged, and older women and men: development and validation. *Behavior Research Methods*. 2010;42(1):351-362.
9. Lundqvist, D., Flykt, A., & Öhman, A. (1998). The Karolinska directed emotional faces (KDEF). *CD ROM from Department of Clinical Neuroscience, Psychology section, Karolinska Institutet,* 91(630), 2-2.
